# Genetic Variants at 1p11.2 and Breast Cancer Risk: A Two-Stage Study in Chinese Women

**DOI:** 10.1371/journal.pone.0021563

**Published:** 2011-06-27

**Authors:** Yue Jiang, Hao Shen, Xiao'an Liu, Juncheng Dai, Guangfu Jin, Zhenzhen Qin, Jiaping Chen, Shui Wang, Xinru Wang, Zhibin Hu, Hongbing Shen

**Affiliations:** 1 State Key Laboratory of Reproductive Medicine, Institute of Toxicology, Nanjing Medical University, Nanjing, China; 2 MOE Key Laboratory of Modern Toxicology, School of Public Health, Nanjing Medical University, Nanjing, China; 3 Section of Clinical Epidemiology, Jiangsu Key Lab of Cancer Biomarkers, Prevention and Treatment, Cancer Center, Nanjing Medical University, Nanjing, China; 4 Department of General Surgery, The First Affiliated Hospital of Nanjing Medical University, Nanjing, China; Vrije Universiteit Medical Center and Center for Neurogenomics and Cognitive Research, The Netherlands

## Abstract

**Background:**

Genome-wide association studies (GWAS) have identified several breast cancer susceptibility loci, and one genetic variant, rs11249433, at 1p11.2 was reported to be associated with breast cancer in European populations. To explore the genetic variants in this region associated with breast cancer in Chinese women, we conducted a two-stage fine-mapping study with a total of 1792 breast cancer cases and 1867 controls.

**Methodology/Principal Findings:**

Seven single nucleotide polymorphisms (SNPs) including rs11249433 in a 277 kb region at 1p11.2 were selected and genotyping was performed by using TaqMan® OpenArray™ Genotyping System for stage 1 samples (878 cases and 900 controls). In stage 2 (914 cases and 967 controls), three SNPs (rs2580520, rs4844616 and rs11249433) were further selected and genotyped for validation. The results showed that one SNP (rs2580520) located at a predicted enhancer region of *SRGAP2* was consistently associated with a significantly increased risk of breast cancer in a recessive genetic model [Odds Ratio (OR)  =  1.66, 95% confidence interval (CI)  =  1.16–2.36 for stage 2 samples; OR  =  1.51, 95% CI  =  1.16–1.97 for combined samples, respectively]. However, no significant association was observed between rs11249433 and breast cancer risk in this Chinese population (dominant genetic model in combined samples: OR  =  1.20, 95% CI  =  0.92–1.57).

**Conclusions/Significance:**

Genotypes of rs2580520 at 1p11.2 suggest that Chinese women may have different breast cancer susceptibility loci, which may contribute to the development of breast cancer in this population.

## Introduction

Breast cancer is one of the most common cancers in the world and becoming the first cancer-related killer to the women. It was reported that the global burden of breast cancer in women was substantial and on the increase, with 1.38 million new cases diagnosed in 2008 [1], [2]. As a developing country, breast cancer incidence had been increasing 20% to 30% in China's urban registries in the past decade [3]. Numerous loci were identified to have significantly association with the higher risk of breast cancer, suggesting an important contribution of inherited factors to breast cancer susceptibility [4]–[7].

Recently, a multistage genome-wide association study (GWAS) of breast cancer identified a new breast cancer susceptible locus at 1p11.2 (the marker SNP: rs11249433) in populations of European descent, which resides in a large linkage disequilibrium (LD) block neighboring *NOTCH2* and *FCGR1B* [4]. Subsequently, another combined analysis of GWAS in breast cancer reported the consistent result based on three GWAS of European descent [8].

1p11.2 has been reported to have the underlying function in mediating the cell growth and differentiation, and is regarded as a region to mediate the intra-chromosomal recombination event [9]. Notch signaling at this region contributes a lot to cell differentiation, survival and proliferation, and any alterations of the pathway may lead to various disorders including human malignancies [10]–[12]. FCGRs as the high affinity IgG required in IgG immune responses may play a crucial role in linking humoral and cellular branches of the immune system [13]. Based on its functional importance, there were several published studies on this gene family with immune disorders and breast cancer [14]–[17].

However, up to now, the association of genetic variants at 1p11.2 with breast cancer risk has not been evaluated in populations of non-European descent. Specially, the minor allele frequency (MAF) of rs11249433 is prominently lower in populations of Chinese (0.022), Japanese (0.011) and African (0.067) than that in population of European descent (0.450), suggesting that there may be a heterogeneity of associations between genetic variants in this region and breast cancer susceptibility among different populations. Thus, to evaluate the role of genetic variants at 1p11.2 with breast cancer susceptibility in Chinese, we performed a fine mapping of approximately 277 kb region at 1p11.2 with a two-stage case-control study including 1,792 breast cancer cases and 1,867 controls.

## Materials and Methods

### Ethics Statement

This study was approved by the institutional review board of Nanjing Medical University. The design and performance of current study involving human subjects were clearly described in a research protocol. All participants were voluntary and would complete the informed consent in written before taking part in this research.

### Study subjects

A total of 1,792 breast cancer cases and 1,867 cancer-free controls were included in this study. All subjects were genetically unrelated ethnic Han Chinese women from Nanjing City and surrounding regions in southeast China. Briefly, breast cancer cases were recruited from the First Affiliated Hospital of Nanjing Medical University, the Cancer Hospital of Jiangsu Province and the Gulou Hospital, Nanjing, China, from January 2004 to April 2010. All the cases were newly diagnosed and histopathologically confirmed without restrictions of age or histological type. Cancer-free women controls were frequency-matched to the cases on age and residential area (urban or rural), and were randomly selected from a cohort of more than 30,000 participants in a community-based screening program for non-infectious diseases conducted from 2004 to 2006 in Jiangsu Province, China. A pretested questionnaire was completed by trained interviewers to collect information on demographic data, menstrual and reproductive history, and environmental exposure history after the informed consent was obtained from the participant. Approximately 5 ml of venous blood was collected from each subject after the interview. The estrogen receptor (ER) and progesterone receptor (PR) status of breast cancer was abstracted from the medical records of patients. In general, 878 cases and 900 controls were randomly selected into the first fine-mapping stage, and the remaining 914 cases and 967 controls formed the validation stage.

### SNP selection

Based on the public HapMap SNP database (phase II Nov 08, on NCBI B36 assembly, dbSNP b126), we selected tagging SNPs for the region approximately 277 kb from 120.7 to 121.1 Mb at 1p11.2 (the LD block where the marker SNP rs11249433 located) with MAF ≥ 0.05 in Chinese Han population (CHB). Six SNPs (rs2580520, rs4844616, rs12033387, rs6600745, rs4259688, rs12743918) were selected based on an r^2^ threshold of 0.8 by the Haploview software. Using UCSC Genome Browser (http://genome.ucsc.edu/cgi-bin/hgGateway), there were two different locations (Feb.2009, GRCh37/hg19, dbSNP 132) of rs2580520, referring to 1p11.2 and 1q32.1. We designed the primers and probes for rs2580520 at 1p11.2. In addition, the previously reported SNP rs11249433 which was significantly associated with breast cancer risk in Caucasian and with MAF of 0.022 in CHB was directly included in our study.

### Genotyping assay

Genomic DNA was isolated from leukocyte pellets of venous blood by proteinase K digestion and followed by phenol–chloroform extraction. For the stage 1 samples, SNPs were genotyped by using the TaqMan® OpenArray™ Genotyping System (Applied Biosystems Inc.). Normalized human DNA samples were loaded and amplified on customized arrays following the manufacturer's instructions. Each 48-sample array chip contained two NTCs (no template controls). The overall call rate was from 97.2% to 99.8% for all 7 SNPs in 878 breast cancer cases and 900 controls. In the validation stage, the SNPs that were significantly associated with breast cancer risk in stage 1 were further genotyped for the stage 2 subjects with 914 breast cancer cases and 967 controls by using the TaqMan assay on ABI PRISM 7900 HT platform (Applied Biosystems Inc.). Approximately equal numbers of case and control samples were assayed in each 384-well plate with two NTCs. Primers and probes were available upon request. Genotyping was performed by blinding the case or control status. Randomly selected 96 duplicates were examined by the two platforms and the coincidence rate was 100%.

### Statistical analyses

The *χ* ^2^ test (for categorical variables) and student *t* test (for continuous variables) were used to analyze the differences in demographic characteristics, selected variables and frequencies of the genotypes between the cases and controls. Odds ratios (ORs) and 95% confidence intervals (CIs) were calculated by using logistic regression model with adjustment for age, age at menarche and menopausal status to estimate the associations between the genotypes and breast cancer risk. Haplotype frequencies were obtained from PHASE 2.1 program based on the observed genotypes. We used CaTS 0.02 to perform the power estimation of two stage association studies. All of the statistical analyses were performed with Statistical Analysis System software (9.1.3; SAS Institute, Cary, NC, USA).

## Results

The distribution of selected characteristics between the overall 1,792 breast cancer cases and 1,867 cancer-free controls in two stages are summarized in Table 1. Compared with the control subjects, the breast cancer cases had a younger age at menarche (*P*<0.0001) and an older age at first live birth (*P*<0.0001). On average, older age at menopause achieved a borderline significance in breast cancer (*P* = 0.051). Among breast cancer subjects, 803 (55.5%) cases were ER positive and 643 (44.5%) were negative. Similarly, 810 (56.1%) cases were positive with PR while 634 (43.1%) were negative.

**Table 1:** Distributions of select variables in breast cancer cases and cancer-free controls.

Variables	Stage 1		Stage 2		Combined		Combined
	case(N = 878)	control(N = 900)	case(N = 914)	control(N = 967)	case(N = 1792)	control(N = 1867)	*P* value
Age, year (mean ± SD)	51.29±11.38	51.47±11.67	50.11±11.36	48.64±12.28	50.69±11.38	50.01±12.07	0.077
Age at menarche, year (mean ± SD)^a^	15.25±1.85	15.85±1.90	15.18±1.96	16.24±1.81	15.21±1.90	16.05±1.86	<0.0001
Age at first live birth, year (mean ± SD)^b^	25.65±3.48	24.90±3.40	25.55±3.16	24.15±2.58	25.60±3.32	24.51±3.03	<0.0001
Age at natural menopause, year (mean ± SD)^c^	49.85±3.18	49.15±4.09	49.71±3.72	49.69±3.56	49.78±3.45	49.42±3.84	0.051
Menopausal status							<0.0001
Premenopausal	416	437	434	530	850	967	
Natural menopause	386	432	379	418	765	850	
Unnatural menopause	58	16	84	9	142	25	
Estrogen receptor (ER)							
Positive	369		434		803		
Negative	321		322		643		
Progesterone receptor (PR)							
Positive	396		414		810		
Negative	294		340		634		

a Age at menarche information was available in 862 breast cancer cases and 896 controls (stage 1), 893 breast cancer cases and 962 controls (stage 2).

b Age at first live birth information was available in 830 breast cancer cases and 872 controls (stage 1), 850 breast cancer cases and 943 controls (stage 2).

c Age at natural menopause information was available in 380 breast cancer cases and 411 controls (stage 1), 363 breast cancer cases and 398 controls (stage 2).

The genotype distributions of the 7 SNPs at 1p11.2 with MAF and their associations with breast cancer risk are showed in Table 2. The observed genotype frequencies for these seven SNPs were all in agreement with Hardy–Weinberg equilibrium in the controls (*P* = 0.08, 0.724, 0.486, 0.189, 0.708, 0.289 and 0.997 for rs2580520, rs4844616, rs12033387, rs11249433, rs6600745, rs4259688 and rs12743918, respectively). In the single locus analyses, two of the seven polymorphisms achieved significant difference in the genotype distributions between cases and controls (*P* = 0.013, 0.014 for rs2582520 and rs4844616, respectively). In stage 1, logistic regression analysis suggested that rs4844616 CT genotype had a 23% reduction of breast cancer risk (OR  =  0.77, 95% CI  =  0.63–0.94, *P* = 0.011), while the combined rs4844616 CT/TT genotypes had a 18% reduction (OR  =  0.82, 95% CI  =  0.68–1.00, *P*  = 0.045), when compared with the CC genotype. Rs2580520GG genotype was associated with a non-significant increased breast cancer risk in the recessive genetic model for stage 1 subjects (OR  =  1.40, 95% CI  =  0.93–2.09, *P*  =  0.106). Besides, the borderline significance of rs11249433 was also observed in genotype distribution (*P* = 0.071), and when CC genotype compared with TT genotype (OR  =  4.29, 95% CI  =  0.90-20.47, *P*  =  0.068). However, there were no obvious evidences of significant associations between other five SNPs (rs12033387, rs6600745, rs4259688, rs12743918) and breast cancer risk.

**Table 2:** Genotype distributions of 1p11.2 and their associations with breast cancer risk.

	Stage 1				Stage 2				Combined				
Genotype	Case	Control	Adjusted OR	Adjusted	Case	Control	Adjusted OR	Adjusted	Case	Control	Adjusted OR	Adjusted	MAF
	**(N = 878)**	**(N = 900)**	**(95% CI)** a	***P*** ** value**	**(N = 914)**	**(N = 967)**	**(95% CI)** a	***P*** ** value**	**(N = 1792)**	**(N = 1867)**	**(95% CI)** a	***P*** ** value**	**Case/Control**
rs2580520													
CC	552	539	1.00	0.013^b^	564	580	1.00	0.000^b^	1116	1119	1.00	<0.0001^b^	
CG	257	308	0.83(0.68,1.03)	0.089	242	307	0.84(0.68,1.04)	0.107	499	615	0.84(0.72,0.97)	0.019	0.233/0.225
GG	65	44	1.31(0.87,1.98)	0.195	100	62	1.56(1.09,2.25)	0.015	165	106	1.42(1.09,1.86)	0.010	
CC/CG			1.00				1.00				1.00		
GG			1.40(0.93,2.09)	0.106			1.66(1.16,2.36)	0.005			1.51(1.16,1.97)	0.002	
rs4844616													
CC	414	381	1.00	0.014^b^	386	425	1.00	0.445^b^	800	806	1.00	0.531^b^	
CT	363	431	0.77(0.63,0.94)	0.011	398	396	1.11(0.91,1.37)	0.309	761	827	0.92(0.80,1.07)	0.279	0.336/0.342
TT	101	84	1.10(0.79,1.53)	0.580	117	136	0.91(0.67,1.23)	0.527	218	220	0.98(0.79,1.23)	0.888	
CT/TT	464	515	0.82(0.68,1.00)	0.045	515	532	1.06(0.87,1.29)	0.549	979	1047	0.94(0.82,1.07)	0.341	
rs12033387													
GG	628	654	1.00	0.762^b^									
AG	228	226	1.03(0.83,1.29)	0.791									0.152/0.144
AA	19	16	1.24(0.62,2.49)	0.536									
rs11249433													
TT	777	822	1.00	0.071^b^	850	909	1.00	0.237^b^	1627	1731	1.00	0.162^b^	
CT	73	68	1.09(0.76,1.55)	0.641	57	50	1.34(0.88,2.03)	0.170	130	118	1.17(0.89,1.53)	0.261	0.042/0.034
CC	9	2	4.29(0.90,20.47)	0.068	0	2	–	0.969	9	4	2.24(0.65,7.65)	0.200	
rs6600745													
GG	259	253	1.00	0.812^b^									
CG	428	445	0.95(0.76,1.18)	0.621									0.461/0.462
CC	190	186	0.99(0.75,1.30)	0.914									
rs4259688													
TT	297	269	1.00	0.131^b^									
CT	399	449	0.82(0.66,1.02)	0.075									0.415/0.439
CC	152	162	0.83(0.63,1.10)	0.199									
rs12743918													
GG	535	510	1.00	0.134^b^									
AG	297	332	0.85(0.69,1.04)	0.105									0.217/0.246
AA	41	54	0.70(0.45,1.08)	0.110									

a Adjusted by age, age at menarche, menopausal status.

b Two-sided χ2 test for difference in frequency distribution of genotypes between cases and controls.

Based on the results of stage 1, two promising SNPs (rs2580520 and rs4844616) that were significantly different of their genotype distributions between the cases and controls were selected for the stage 2 validation. In addition, we also select rs11249433 for further validation to avoid the underestimation due to low MAF and limited sample set of stage 1 (Table 2). We found the association with rs4844616 was not validated (CT/TT vs. CC: OR  =  1.06, 95% CI  =  0.87–1.29, *P*  =  0.549 for stage 2 samples; OR  =  0.94, 95% CI  =  0.82–1.07, *P* = 0.341 for the combined samples). However, no significant difference was observed for rs11249433 genotypes between cases and controls (CT *vs.* TT: OR  =  1.17, 95% CI  =  0.89–1.53, *P* = 0.261 for the combined samples; CC *vs.* TT: OR  =  2.24, 95% CI  =  0.65–7.65, *P* = 0.200 for combined samples). Taking account of multiple comparisons, we also used bonferroni adjustment to adjust the second stage results, and *P* = 7.1×10^−3^ (0.05/6) was considered as the significance threshold. Among all SNPs, only rs2580520 still had a significantly increased risk of breast cancer in the recessive genetic model (OR  =  1.66, 95% CI  =  1.16–2.36, *P* = 0.005 for stage 2 samples). Furthermore, Haplotype analysis was performed for these three polymorphisms. As shown in Table 3, three common haplotypes were identified to account for > 90% of all conjectural haplotypes. Compared with the most common haplotype CCT, no single haplotype was associated with the risk of breast cancer.

**Table 3:** Haplotype Analysis of the Identified 3 SNPs.

Haplotype^a^	Cases(n = 1792)	Controls(n = 1867)	OR (95% CI)
CCT	1043(58.2%)	1103(59.1%)	1.00
GTT	314(17.5%)	351(18.8%)	1.06(0.88,1.26)
CTT	274(15.3%)	284(15.2%)	0.98(0.81,1.19)
Others	161(9.0%)	129(6.9%)	0.76(0.59,0.98)

a In the order of rs2580520, rs484416 and rs11249433.

In the stratified analysis, the association with rs2580520 in the recessive genetic model was also evident among premenopausal women (OR  =  1.66, 95% CI  =  1.15–2.39) and women with older menarche age (OR  =  1.54, 95% CI  =  1.13–2.11), and among the cases with ER positive (OR  =  1.62, 95% CI  =  1.17–2.23) or PR positive (OR  =  1.61, 95% CI  =  1.17–2.21). Nevertheless, no heterogeneity was observed between the stratified subgroups (Table 4).

**Table 4:** Stratified analysis on the associations between rs2580520 and risk of breast cancer.

Characteristics	Case			Control			Recessive model		
	CC(%)	CG(%)	GG(%)	CC(%)	CG(%)	GG(%)	OR(95%CI)^a^	*P*	*P* b
Age									
<50	563(63.2)	248(27.9)	79(8.9)	605(61.9)	312(31.9)	61(6.2)	1.53(1.06,2.20)	0.043	0.668
≥50	553(62.1)	251(28.2)	86(9.7)	514(59.6)	303(35.2)	45(5.2)	1.63(1.10,2.41)	0.014	
Menopausal status									
Premenopausal	526(62.1)	242(28.6)	79(9.3)	589(61.4)	314(32.8)	56(5.8)	1.66(1.15,2.39)	0.007	0.520
Postmenopausal^c^	474(62.5)	218(28.7)	67(8.8)	499(59.8)	288(34.5)	48(5.7)	1.39(0.93,2.06)	0.107	
Age at menarche									
<15	428(62.6)	191(27.9)	65(9.5)	234(59.9)	133(34.0)	24(6.1)	1.63(1.00,2.66)	0.050	0.848
≥15	669(63.1)	302(28.5)	89(8.4)	879(61.0)	480(33.3)	82(5.7)	1.54(1.13,2.11)	0.007	
Age at first live birth									
<25	386(63.0)	165(26.9)	62(10.1)	521(57.3)	333(36.6)	55(6.1)	1.70(1.15,2.51)	0.008	0.467
≥25	661(62.5)	311(29.5)	84(8.0)	569(64.6)	263(29.8)	49(5.6)	1.39(0.95,2.02)	0.091	
ER status									
ER+	505(62.3)	215(26.9)	78(9.8)				1.62(1.17,2.23)	0.003	0.568
ER-	394(61.7)	190(29.7)	55(8.6)				1.41(0.99,2.00)	0.057	
PR status									
PR+	503(62.3)	225(27.9)	79(9.8)				1.61(1.17,2.21)	0.004	0.585
PR-	392(62.4)	182(29.0)	54(8.6)				1.41(0.99,2.01)	0.055	

a Adjusted by age, age at menarche, menopausal status where appropriate.

b *p* for heterogeneity.

c Postmenopausal status for natural menopause.

## Discussion

To our knowledge, this is the first fine-mapping study in the Chinese population to assess the association of polymorphisms at 1p11.2 with breast cancer risk. We evaluated one previously reported SNP (rs11249433) and 6 common tagging SNPs (rs2580520, rs4844616, rs12033387, rs660745, rs4259688 and rs12743918) at 1p11.2 by fine mapping a 277 kb LD block. We identified one SNP (rs2580520) as a marker SNP at this region that was significantly associated with breast cancer risk in Chinese.

Previous studies in Caucasian populations on 1p11.2 and breast cancer susceptibility identified the marker SNP of rs11249433. This SNP was 568kb to the transcription start site of *NOTCH2*, a gene associated with insulin release, insulin sensitivity, obesity and type 2 diabetes [18], [19]. As NOTCH family members play a role in a variety of developmental processes by controlling cell fate decisions, more and more studies follow closely on the NOTCH signaling pathway as one of the underlying mechanisms [10] and candidate therapeutic targets for breast cancer [20]. However, the SNP rs11249433 was heterogeneous among different ethnics, with a common frequency of 0.425 in Caucasians and very low frequency of 0.022 in CHB HapMap and 0.040 in our control subjects. Unfortunately, because of the low frequency of rs11249433 that leads to the low power (approximately 31%), we did not find a significant association of this SNP with susceptibility of breast cancer in this Chinese population (CT vs. TT: OR  =  1.17, 95% CI  =  0.89–1.53, *P*  =  0.261, CC vs. TT: OR  =  2.24, 95% CI  =  0.65–7.65, *P* = 0.200 for combined samples). In fact, the heterozygote OR from combined stage is highly consistent with of Thomas's study [4]. Therefore, we cannot exclude the limitation of power may hold back the statistically significant to the true association.

**Figure 1:**
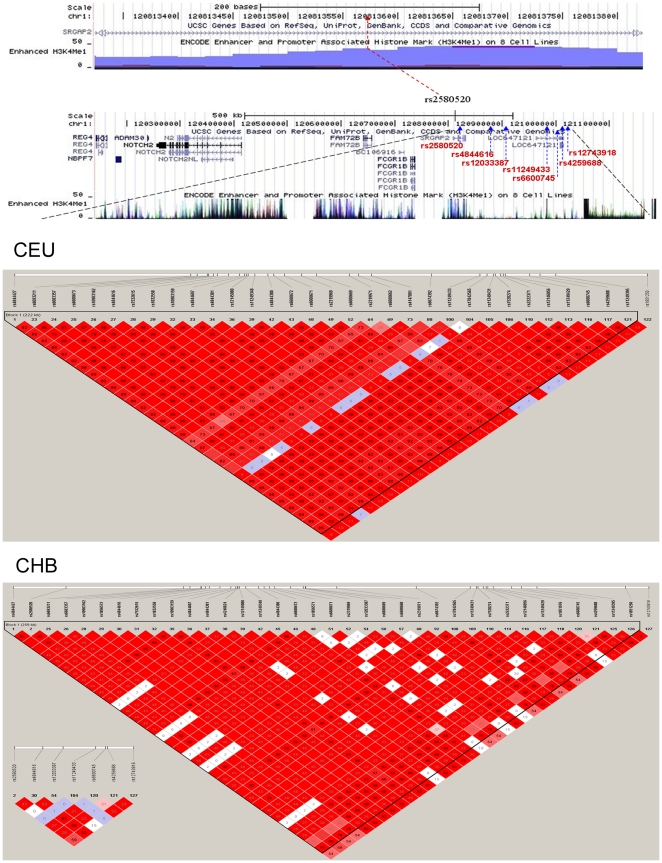
Overview of the LD block and the new finding marker SNP (rs2580520) in our study for breast cancer susceptibility. The upper panel shows a view of the genomic region of rs2580520 (in a predicted H3K4Me1 enhancer region) (120,813,325-120,813,825) from the UCSC browser Build 36 assembly (hg18). The following one shows the analyzed linkage disequilibrium (LD) block, its flanking region, related genes and predicted enhancers, marking with the seven SNPs. The lower panel shows the LD plot (120,791,177 - 121,068,797) of European descent and Chinese descent [the block was defined by the confidence intervals (Gabriel et al., 2002)]. The lower left thumbnail shows the r^2^ vaule between seven SNPs in Chinese descent.Several limitations were inherent in this study. First, the power was relatively low due to the limited sample size and low MAF of the rs11249433 SNP. Second, the biological function of the significant SNP rs2580520 is unclear in the development of breast cancer. Therefore, additional large scale population-based studies will be needed to validate the findings in our study and functional characterization to explore the underline mechanisms will be also required.

With a two-stage fine mapping study at 1p11.2, we identified a marker SNP rs2580520 in Chinese. Rs2580520 was in low LD (pairwise r^2^  =  0.005, Fig. 1) with rs11249433 in Chinese population and no LD in European population (pairwise r^2^  =  0.000). The MAF of rs2580520 was common in Chinese (0.178) but extremely rare in European population (0.000), suggesting the population specificity. This newly-identified marker SNP is located in the intron of *SRGAP2* (SLIT-ROBO Rho GTPase activating protein 2) that has been proved to negatively regulate neuronal migration and induce neurite outgrowth [21]. Using G2SBC (Genes-to-Systems Breast Cancer) database (http://www.itb.cnr.it/breastcancer/index.html), we found the *SRGAP2* expression was up regulated in multiple breast cancer cells. However, the exact mechanism of the SRGAP2 in the development of breast cancer still needs further investigation. As labeled in Figure 1, rs2580520 was located in an enhancer region but far from the *NOTCH2* gene (about 400kb to the transcription start site, UCSC genome browser database, Build 36 assembly, hg18). It is recognized that enhancer can increase the efficiency of transcription of the specific genes even from far distance. Based on the position of rs2580520, we conjectured that this SNP may regulate the upstream genes of transcription starts by enhancer, such as *FCGR1B* or *NOTCH2. NOTCH2* expression was high in well-differentiated tumours but low in poor-differentiated tumours which might play a tumour-suppressive role in human breast cancer [11]. The direct relation between *FCGR1B* and breast cancer was not reported, but FCGR gene family was proved to do with breast cancer risk as mentioned above [17]. Additionally, the position of rs2580520 at pericentromeric regions may alter gene expression during cellular differentiation or reprogramming, and therefore may promote the normal cells into abnormal growth and differentiation which eventually lead to tumors. Together, our results support chromosome 1p11.2 as a susceptibility region for breast cancer and emphasize the difference in genetic markers among different ethnic populations [22].

In summary, our present results provide evidence that Chinese women may have different breast cancer susceptibility loci at 1p11.2 and rs2580520 in this region was associated with genetic susceptibility of breast cancer in Chinese population.
